# Exploring the long-term colonisation and persistence of probiotic-prophylaxis species on the gut microbiome of preterm infants: a pilot study

**DOI:** 10.1007/s00431-022-04548-y

**Published:** 2022-07-07

**Authors:** Jacob A. F. Westaway, Roger Huerlimann, Yoga Kandasamy, Catherine M. Miller, Robert Norton, David Watson, Sandra Infante-Vilamil, Donna Rudd

**Affiliations:** 1 College of Public Health, Medical and Veterinary Science, James Cook University, 1/14-88 McGregor Road, Smithfield, QLD 4878 Australia; 2 Centre for Tropical Bioinformatics and Molecular Biology, James Cook University, 1 James Cook Dr, Douglas, QLD 4811 Australia; 3 Marine Climate Change Unit, Okinawa Institute of Science and Technology (OIST), 1919-1 Tancha, Onna-son, Okinawa, 904-0495 Japan; 4 College of Science and Engineering, James Cook University, 1 James Cook Dr, Douglas, QLD 4811 Australia; 5 College of Public Health, Medical and Veterinary Science, James Cook University, 1 James Cook Dr, Douglas, QLD 4811 Australia; 6 Neonatology, Townsville University Hospital, 100 Angus Smith Dr, Douglas, QLD 4814 Australia; 7 Microbiology, Pathology Queensland, 100 Angus Smith Dr, Douglas, QLD 4814 Australia; 8 Maternal-Fetal Medicine, Townsville University Hospital, 100 Angus Smith Dr, Douglas, QLD 4814 Australia; 9 Faculty of Medicine, University of Queensland, Brisbane, QLD Australia; 10 Menzies School of Health Research, Building 58, John Mathews Building, Royal Darwin Hospital Campus, Casuarina, NT 0810 Australia

**Keywords:** Preterm infant, Neonatal, Microbiome, Probiotics, NICU & metagenomics

## Abstract

Preterm infants suffer from a higher incidence of acute diseases such as necrotising enterocolitis and sepsis. This risk can be mitigated through probiotic prophylaxis during admission. This reduction in risk is likely the result of acute modulation of the gut microbiome induced by probiotic species, which has been observed to occur up until discharge. We aimed to determine if this modulation, and the associated probiotic species, persisted beyond discharge. We conducted both a cross-sectional analysis (*n* = 18), at ~ 18 months of age, and a longitudinal analysis (*n* = 6), from admission to 18 months of the gut microbiome of preterm infants using both shotgun metagenomics and 16S rRNA profiling respectively. The 16S amplicon sequencing revealed that the microbial composition of the probiotic-supplemented infants changed dramatically over time, stabilising at discharge. However, species from the probiotic Infloran^®^, as well as positive modulatory effects previously associated with supplementation, do not appear to persist beyond discharge and once prophylaxis has stopped.

*Conclusions*: Although differences exist between supplemented and non-supplemented groups, the implications of these differences remain unclear. Additionally, despite a lack of long-term colonisation, the presence of probiotics during early neonatal life may still have modulatory effects on the microbiome assembly and immune system training.

**Table:** 

**What is Known:** *• Evidence suggests modulation of the microbiome occurs during probiotic prophylaxis, which may support key taxa that exert positive immunological benefits.* • *Some evidence suggests that this modulation can persist post-prophylaxis.*	
**What is New:** *• We present support for long-term modulation in association with probiotic prophylaxis in a cohort of infants from North Queensland Australia.* • *We also observed limited persistence of the probiotic species post-discharge.*

**Supplementary Information:**

The online version contains supplementary material available at 10.1007/s00431-022-04548-y.

## Introduction

Preterm birth, defined by the World Health Organisation as < 37-week gestation [1], disrupts gut microbiome development [2]. The resulting preterm microbiome is characterised by low diversity and commensal microbe abundance, in combination with a greater number of pathogens [3, 4]. This characteristic preterm microbiome has been linked to increased disease burden in these infants [5]. This includes acute diseases like necrotising enterocolitis (NEC) and late-onset sepsis (LOS), and chronic diseases like asthma and both types 1 and 2 diabetes, all of which have been linked to the microbiome [6]. However, probiotic prophylaxis can mitigate the risk of these acute diseases [7]. As a result, probiotic prophylaxis has now become the standard of care for the most premature (< 32-week gestation) and small for gestational age infants (< 1500 g) in neonatal intensive care units (NICUs) across Australia.

Probiotic prophylaxis has been demonstrated to mitigate and treat several infectious and non-infectious diseases through modulation of the gut microbiome [8]. Probiotic supplementation has been effective in mitigating a number of infections including, *Helicobacter pylori* infection [9], rotavirus infection [10], obesity [11] and allergies [12]. In pre-term infants, probiotics have been shown to reduce the incidence of both NEC and LOS, with the benefits likely stemming from changes in the microbiome afforded by the presence of probiotic strains [8]. These strains, specifically from *Bifidobacterium* and *Lactobacillus*, have been shown to contribute to a *Bifidobacterium*-dominated microbiome, which, in turn, can positively modulate immune system activity and development [6, 13]. Although acute benefits of prophylactic supplementation have been noted in the most premature infants, data on both the long-term impact of probiotic supplementation and on non-supplemented infants remains sparse.

Certain probiotic species have been shown to persist beyond discharge [14], possibly continuing to exert positive effects on the development of the infant gut microbiome. However, this observation of probiotic persistence is not consistent [14, 15], and as infants have been shown to cluster in their microbial populations by NICU [16], caution should be taken when extrapolating from these single unit studies to another unit. We have demonstrated in a previous study that probiotic-prophylaxis had a significant positive modulatory effect on very-preterm infants over the course of their hospital admission [17]. We observed greater diversity in the gut microbiome of probiotic-supplemented preterm infants, relative to those not supplemented, at discharge from the hospital, suggesting that preterm infants who fall outside the criteria for probiotic prophylaxis (defined as < 32-week gestation and/or < 1500 g) may be missing out on the positive modulatory effects for healthy gut microbiome development. Our aim for this study was to investigate if these differences persist following discharge up to 1.5–2 years of age and conduct both a cross-sectional and longitudinal analysis of the gut microbiome of these preterm infants, using both shotgun metagenomics and 16S rRNA profiling. We were particularly interested to determine if the probiotic species, specifically *L. acidophilus* and *B. bifidum,* were persisting in the gut long-term. In addition, to determine if probiotics had a lasting modulatory effect on microbiome development, we compared these probiotic-supplemented infants to a group of infants who were born into the same nursery but did not receive probiotic supplementation. Lastly, we combined a subset of these newly acquired samples with data collected previously, to conduct a longitudinal examination of probiotic-supplemented infants.

## Methods

### Study design

This observational study involves both a longitudinal and cross-sectional component. As the main objective of this project was to examine if probiotic prophylaxis during admission has a lasting effect, we performed a cross-sectional analysis of 18 infants using shotgun metagenomics and compared those who had received probiotics against those who had not. As previously mentioned, a subset of this cohort (*n* = 6) had samples collected as part of an earlier study, and so we also performed a longitudinal analysis of these probiotic-supplemented infants using 16S rRNA amplicon sequencing, as this was the technique used previously.

### Study population

A combination of 16S rRNA gene amplicon and shotgun metagenomic sequencing was used to characterise the faecal microbiome of preterm infants from North Queensland (NQLD), Australia. This region of Australia is disproportionately burdened by preterm birth and low birth weight [18], and its large indigenous population is more likely to experience prematurity relative to other Australians (13%), representing one in ten preterm births in Queensland [18]. The burden of preterm birth in NQLD, which has increased 5% over the last decade [18], places significant stress on the families and healthcare system in this region of Australia.

Infants recruited were previously admitted to the Townsville University Hospital’s (TUH) Neonatal Intensive Care Unit (NICU) and Special Care Nurseries (SCN). The TUH NICU is the only level six tertiary referral unit in NQLD, which is a specialised unit for dealing with complex pregnancies. The criteria for probiotic prophylaxis at the TUH NICU dictate that all high-risk preterm infants (defined as < 32-week gestation and/or < 1500 g) receive Infloran^®^ [19], containing *Lactobacillus acidophilus* (1 × 10^9^ CFU) and *Bifidobacterium bifidum* (1 × 10^9^ CFU) on a daily basis, from the first day of feeding to > 34–36-week gestation. Inclusion criteria for the cohort included born < 32-week gestation and previously admitted to the NICU at the TUH for the probiotic group and > 32 weeks and admitted to the SCN at the TUH. The exclusion criteria were no parental consent, born > 32 weeks and contraindication to enteral feeds for the probiotic group, and no parental consent for the non-supplemented group. Ethics was obtained from the Townsville Hospital and Health Service Human Research Ethics, (HREC/QTHS/65181 and HREC/17/QTHS/7). Informed consent was obtained from parents/legal guardians of all subjects through the signing of a Parental Information Sheet and Consent Form (PICF), which can be found in the Supplementary material. 

### Recruitment and sample collection

Recruitment of infants previously admitted to the hospital and who were now between 18 months and 2 years of age was conducted by a neonatal nurse, who works in the nurseries, between January and August of 2021. Parents/guardians of previously admitted preterm infants were contacted via the phone, and upon verbal approval, mailed out a PICF and a collection kit. The collection kit included:

- OMNIgene^®^ GUT all in one system
- Paid return postal package
- Detailed instructions on sample collection and postage
- Absorbent material and leak proof biohazard bag for postage requirements
- Questionnaire

The samples of recruited infants were stored in the OMNIgene^®^ GUT collection tube, and once mailed back to the research team, the tubes were stored at − 80 °C, as recommended by the manufacturer.

### Collection of metadata

Both clinical (during admission) and post-discharge metadata were also collected (Table 1). For the clinical data, this included both maternal — antenatal antibiotics, chorioamnionitis (clinically diagnosed), preeclampsia (clinically diagnosed), and diabetes (type 1 & 2), and infant data — mode of delivery (vaginal birth versus caesarean section), diet, gestation, NEC (stage 2 or greater), sepsis (confirmed through culture), neonatal antibiotics and retinopathy of prematurity (ROP) (stage 1 or greater). The post-discharge information was collected through the previously mentioned brief questionnaire.

### 16S rRNA short amplicon sequencing

We used the Bioline ISOLATE Fecal DNA Kit for DNA extraction [20]. Modifications were made in consultation with the manufacturer to optimise DNA yield and included increased beta-mercaptoethanol from 0.5 to 1% (increasing DNA solubility and reducing secondary structure formation), addition of an extra wash step (improving purity) and decreased elution buffer volume from 100 to 50 μl (increasing final DNA concentration). After consultation with the manufacturer, 150 µl was chosen for the initial sample volume, in place of the usual 150 µg required by the kit, for compatibility with the OMNIgene^®^ GUT kit. The Illumina metagenomics library preparation protocol was used for library preparation [21], using the Index Kit v2 C [22] and Platinum™ SuperFi™ PCR Master Mix [23]. Sequencing was performed on the Illumina MiSeq system using the MiSeq Reagent Kit V3 [22], targeting the V3 and V4 regions with the S-D-Bact-0431-b-S-17/S-D-Bact-0785-a-A-21primer combination [21]. Both the pre-analytical bioinformatics and statistical analyses were conducted in *R Studio* Version 3.6.1 [24] with a pipeline adapted from our previous work [25], which can be found in the Supplementary material. *DADA2* [26] was used for quality filtering and trimming, demultiplexing, denoising and taxonomic assignment (SILVA Database). In addition, *microDecon* [27] was used to remove homogenous contamination from samples using extraction blanks.

### Admission and discharge samples for longitudinal analyses

Data for a subset of individuals that had samples collected at both admission and just prior to discharge were obtained from previous work. As we recruited from the same hospital, a small subset (*n* = 6) had samples collected at previous time points, allowing us to make comparisons across these three time points, within the probiotic-supplemented group. However, it should be noted that for one infant, we did not receive an admission sample. The recruitment, collection and sequencing protocols are as previously described [25].

### Shotgun metagenomics

The shotgun metagenomics was performed by Microba Life Sciences [28]. Once samples had DNA extracted for 16S rRNA gene amplicon sequencing, the samples were then again stored at − 80 °C, and, soon after, shipped to Microba on dry ice. Sequencing was conducted on the Illumina NovaSeq6000 system with 300 bp, paired-end reads. This workflow was completed using Microba’s patented metagenomics analysis platform (MAP), which includes the Microba Genome Database, the Microba Community Profiler and the Microba Gene and Pathway Profiler [28]. The MAP produces taxonomic and functional profiles.

### Statistical analysis

For both the 16S rRNA short amplicon sequencing and shotgun metagenomics, we assessed beta diversity, alpha diversity and taxonomic abundance using mixed effects models. For beta diversity comparisons, we performed both PERMANOVA and *EnvFit* analyses from the *Vegan* package [29], which compare the differences in the centroids relative to total variation. Both analyses were applied to Bray–Curtis dissimilarity matrices [30] based on data normalised through total sum scaling (TSS) [31]. The significance was based on 10,000 permutations and was transformed based on the Benjamini-Hochberg (BH) procedure [32].

For alpha diversity comparisons, we performed generalised linear mixed effects models. The generalised linear mixed effects regression models were created using the package *lme4* [33]. Shannon diversity was calculated at the ASV level, on normalised data (TSS), and continuous predictors were scaled and centred. Multicollinearity was assessed using the *AED* package [34], and significance was using an analysis of deviance (type II Wald chi-square test) from the *car* package [35]. This was followed by subsequent post-hoc pairwise Tukey comparisons, to correct for multiple comparisons, using the *emmeans* package [36].

*DESeq2* [37], which uses a negative binomial generalised linear model and variance stabilising transformation, was used for comparing taxonomic abundances between groups. For the 16S rRNA short amplicon sequencing, taxa were agglomerated at the genus level, due to the limited taxonomic depth of 16S-target technologies. A Wald test with the BH multiple inference correction was performed to obtain taxa that were significantly differentially abundant. The pre-analytical bioinformatics and statistical analyses can be found in the GitHub link in the Supplementary material. The results for each of the statistical analyses can be found in the Supplementary File 1.

## Results

### Changes in the gut microbiome of probiotic-supplemented infants over time

**Fig. 1:**
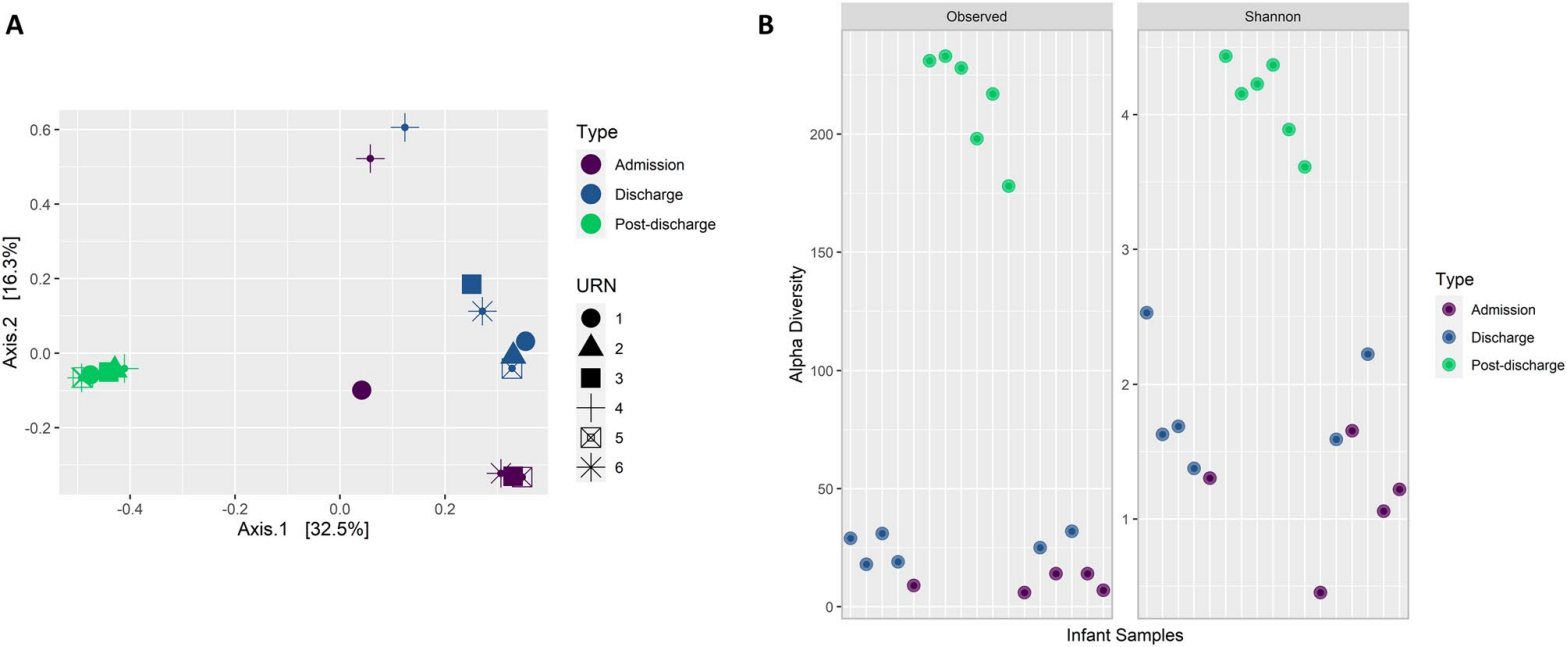
**A** Principal coordinate analysis plot based on Bray–Curtis distances using ASV level taxonomy obtained through 16S rRNA short amplicons sequencing demonstrating the changes in gut microbial composition for the six infants tracked over time, with significant (*p* < 0.01) clustering of samples. **B** Dot plot representing the time-based increases in alpha diversity metrics for the same six infants tracked over time and based on transformed ASV level taxa (16S amplicon sequencing), both observed (richness) and the Shannon Index, where pairwise comparisons found significant differences between admission and discharge samples (*p* = 0.01), admission and post-discharge (*p* < 0.0001) and discharge and post-discharge (*p* < 0.0001)

**Fig. 2:**
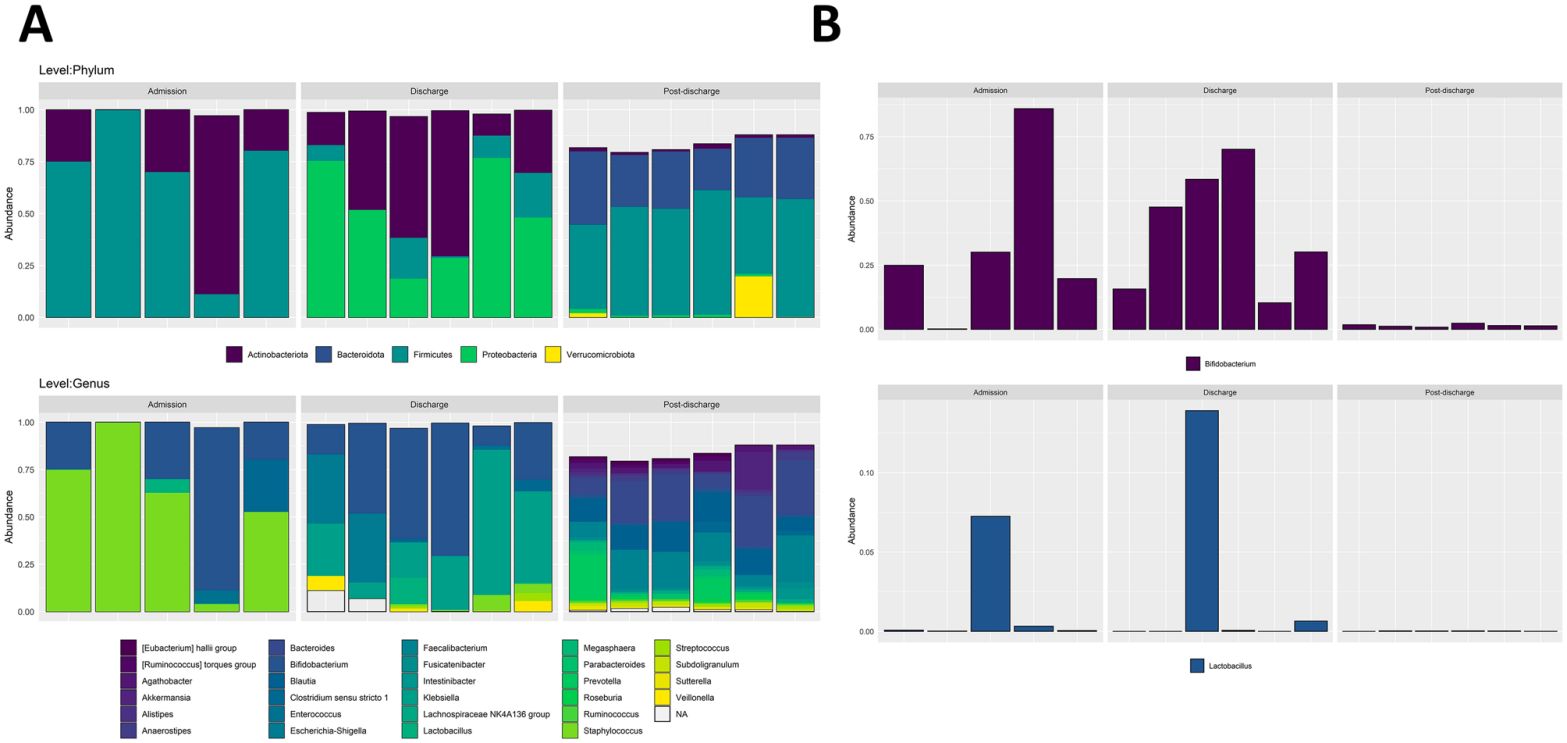
**A** Changes in the proportions of taxa for the six infants tracked over time at both the phylum and genus levels (16S amplicon sequencing) across admission, just prior to discharge and post discharge, describing the significant (*p* < 0.0001) reduction in Bifidobacterium abundance post-discharge relative to the first two time points. **B** Changes in the proportions of both Bifidobacterium and Lactobacillus for the six infants tracked (16S amplicon sequencing) across admission, just prior to discharge, and post discharge, using 16S amplicon sequencing

The data from the 16S amplicon sequencing revealed that the microbiome composition of probiotic-supplemented infants changed dramatically over time, stabilising at discharge (Fig. 1A). Samples clustered significantly by the sampling time based on their taxonomic composition (Fig. 1A, p < *0.01*), coupled with a significant increase in alpha diversity post-discharge (Fig. 1B, *admission and post-discharge: p* < *0.0001, and discharge and post-discharge: p* < *0.0001*), as taxa continued to colonise. The composition is dominated early on by the phylum Firmicutes, followed by Proteobacteria and Actinobacteriota at discharge, and then Bacteroidota and Firmicutes post-discharge (Fig. 2A). At the genus level, we see *Streptococcus*, a facultative anaerobe, dominating at admission, followed by *Bifidobacterium* at discharge, and then maturation to a more diverse ecosystem post-discharge. The changes in *Streptococcus* over time were significant (*p* < *0.01*), with it being in significantly greater abundance early on, compared with both discharge (*p* < *0.001*) and post-discharge (*p* < *0.01*) samples. A similar pattern was seen for *Bifidobacterium*, which was in significantly greater abundance at both admission (*p* < *0.001*) and discharge (*p* < *0.001*), relative to post-discharge samples.

### Persistence of probiotic species present at discharge up to 2 years of age

*Bifidobacterium* was present at admission, discharge and post-discharge; however, *Lactobacillus* was sparse at all time points (Fig. 2B). *Bifidobacterium* was at its greatest abundance at discharge, and thus, towards the end of supplementation. However, despite being present across all infants, there was a significant reduction in its abundance post-discharge. Using shotgun metagenomics, we observed that what remains of the genus post-discharge is mostly other *Bifidobacterium* species, with *B. bifidum* only present in 4/14 infants (Table 2). The other *Bifidobacterium* species present were *B. adolescentis*, *B. animalis*, *B. breve* and *B. longum*, with *B. longum* as the most common and *B. breve* as having the greatest mean relative abundance. *L. acidophilus* was also not present post-discharge (Table 2). The only two species from this genus present post-discharge were *L. paracsei* and *L. rhmanosus*. The lack of long-term colonisation with *L. acidophilus* is consistent with previous work; however, *B. bifidum* has been observed to persist post-discharge at ~ 58 weeks [14]. The scarcity of probiotic species present in our cohort of infants suggests transient colonisation.

**Table 1:** Demographic/clinical data of study population used for both the 16S metabarcoding and shotgun metagenomics

**Variables**	**Levels**	***n***
**Categorical variables**		
**Probiotics during admission**	Yes	14
	No	4
**Probiotics post-discharge**	Yes	9
	No	9
**Diet during admission**	Combination	9
	Breastmilk	9
**Diet post-discharge**	Combination	15
	Breastmilk	3
**Mode of birth**	Vaginal	5
	Caesarean	13
**NEC**	Yes	0
	No	18
**Sepsis**	Yes	0
	No	18
**Antenatal antibiotics**	Yes	7
	No	11
**Neonatal antibiotics**	Yes	17
	No	1
**Chorioamnionitis**	Yes	2
	No	16
**Preeclampsia**	Yes	2
	No	0
**Maternal diabetes**	Yes	1
	No	17
**Continuous variables**		
**Variable**	**mean/median**	
**Gestational age at birth**	30.0 $\pm \;1.3$

**Table 2:** The number of infants that had species belonging to Bifidobacterium or Lactobacillus present post-discharge, determined through shotgun metagenomic sequencing

**Genus**	**Species**	**Supplemented infants (*n*** **= 14)**
***Bifidobacterium***	*Bifidum*	*4*
	*Adolescentis*	*5*
	*Animalis*	*9*
	*Breve*	*5*
	*Longum*	*11*
***Lactobacillus***	*Acidophilus*	*0*
	*Paracsei*	*2*
	*Rhmanosus*	*7*

### Comparison of probiotic-supplemented infants and non-supplemented infants

**Fig. 3:**
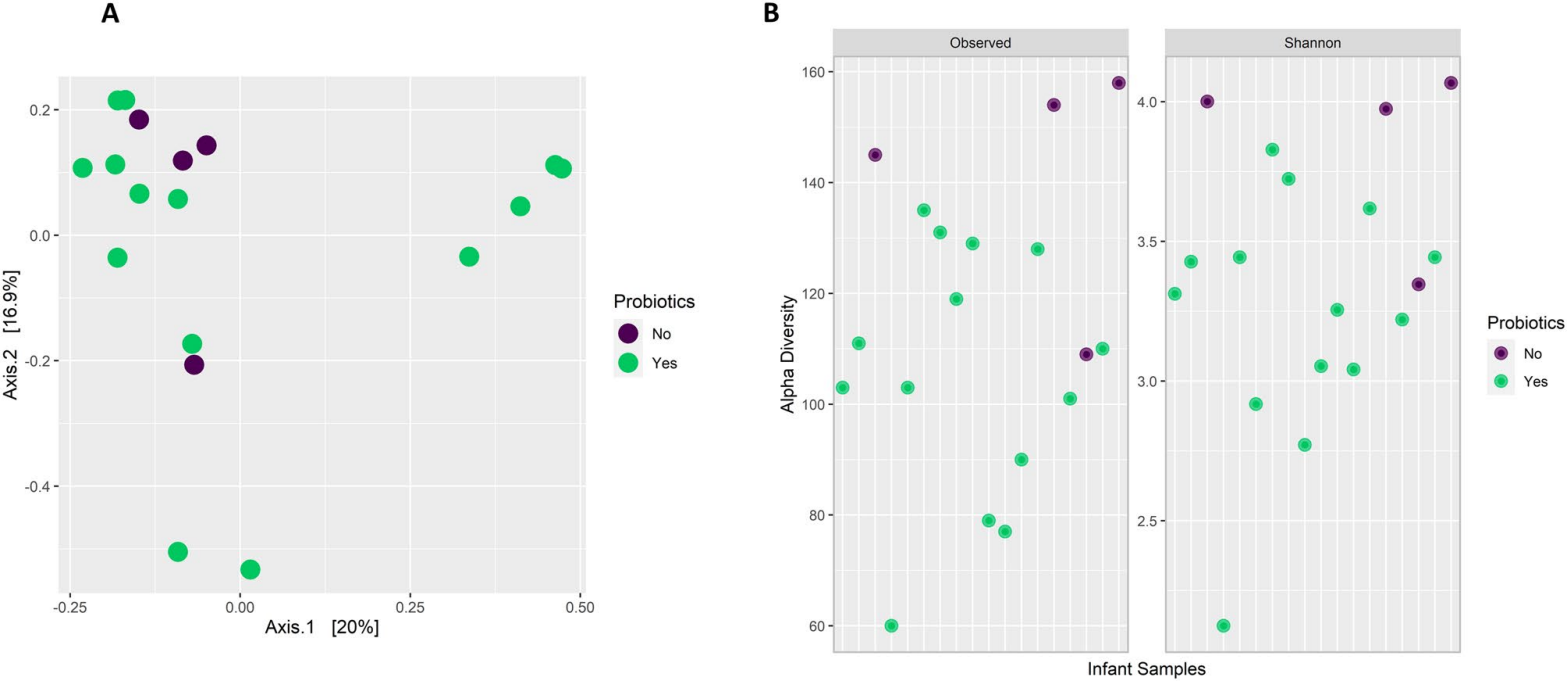
**A** principal coordinate analysis plot based on Bray–Curtis distances exploring the clustering of samples post-discharge by probiotic-supplementation (coloured) using species level taxonomy obtained through shotgun metagenomics. **B** Dot plots describing the significant difference in alpha diversity metrics post-discharge, both observed (richness) (*p* < 0.05) and the Shannon Index (*p* < 0.05), between probiotic supplementation groups and obtained through shotgun metagenomics

**Fig. 4:**
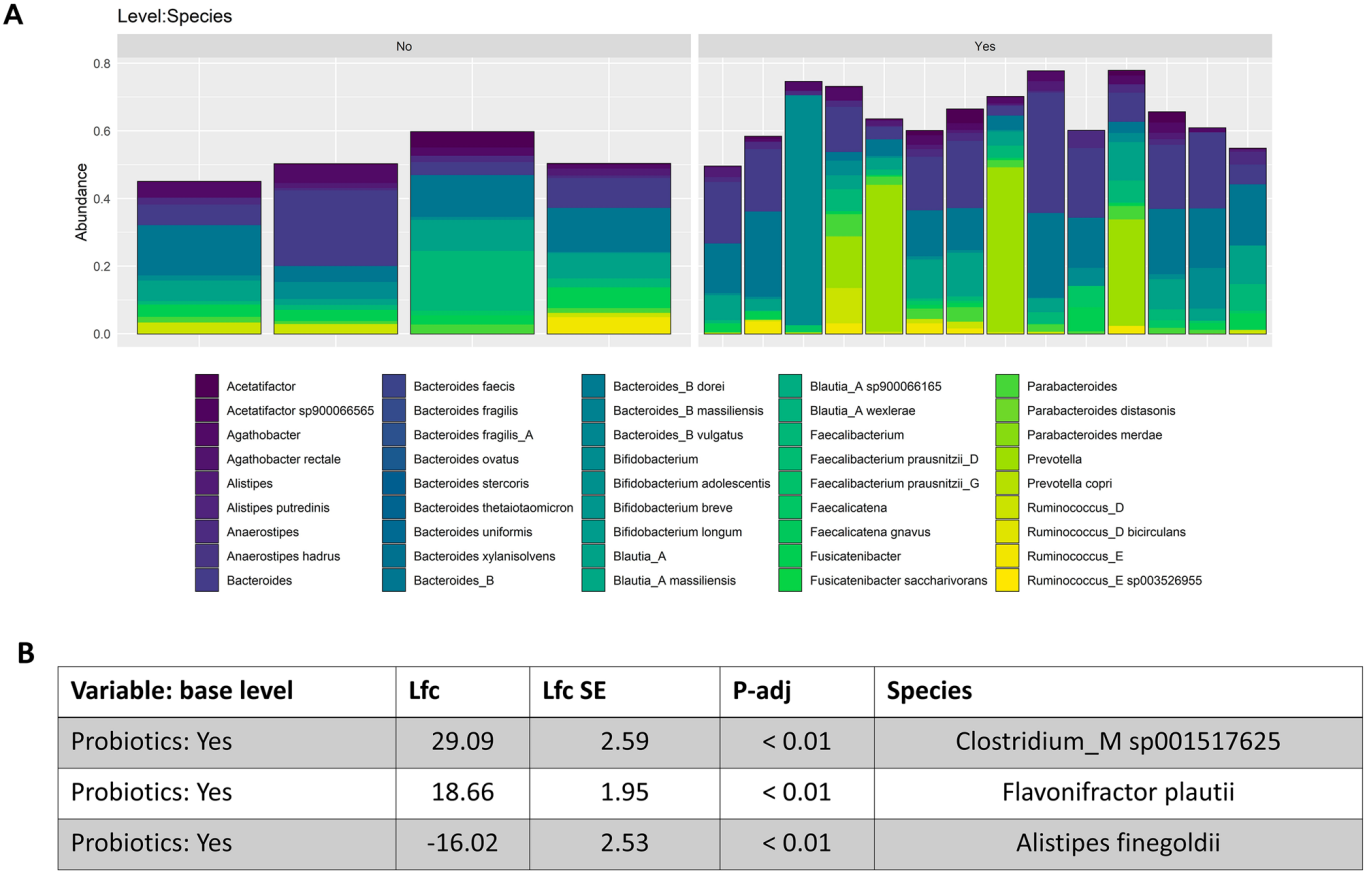
**A** Bar plots comparing the relative distribution of the top 30 most abundant species identified through shotgun metagenomics, and across individuals and between probiotic-supplementation status. **B** results of Wald-test on the probiotic-supplementation comparison from DESeq2 mixed effects modelling, that also accounted for diet, on species level taxonomy obtained through shotgun metagenomics

Previously identified positive modulation of the gut microbiome associated with probiotic prophylaxis during hospital admission does not persist at 18 months to 2 years post-supplementation; however, several other associations were observed. Overall community composition did not differ significantly between those who received probiotic prophylaxis and those who did not (Fig. 3A, *PERMANOVA: p* = *0.4, envfit: p* = *0.88*). However, differences in several taxa were oberved (Fig. 4). Specifically, we observed greater abundance of *Clostridium_M sp001517625* (*p* < *0.01*) and *Flavinofractor plauti* (*p* < *0.01*), in combination with lower abundances of *Alistipes finegoldi* (*p* < *0.01*), in those that received probiotic prophylaxis. *Clostridium_M sp001517625* (11/14 infants) and *Flavonifractor plauti* (13/14 infants) were only observed in the probiotic group. In contrast, *Alistipes finegoldi* was only found in half of those supplemented, but all of those who did not receive probiotics. Lastly, counter to what was expected, alpha diversity, both richness (*p* < *0.05*) and the Shannon Index (*p* < *0.05*) were significantly lower in those infants supplemented with probiotics (Fig. 3B). However, it is unclear whether this associated modulation is a result of probiotic prophylaxis or evidence of an inability of probiotics to exhibit lasting modulation beyond the supplementation period.

## Discussion

We have previously described the short-term positive modulatory impact that probiotic supplementation with Infloran^®^ can have on the developing gut microbiome of preterm infants during their NICU admission [17], supporting an increased diversity and colonisation with beneficial taxa, such as *Bifidobacterium*. This study sets out to investigate whether colonisation by these probiotic species affected the development of a healthy microbiome at 18 months to 2 years post discharge. We observed significant changes in the microbiome of supplemented infants over time, with increases in alpha diversity and dynamic changes in taxonomic abundance, culminating in a reduction in heterogeneity between samples and stabilisation of the microbiome. However, these dynamic changes were coupled with a significant reduction in probiotic species. Several studies report persistence of the probiotic species in the faeces of preterm infants up to the time of hospital discharge [38–41], yet evidence for long-term colonisation with probiotic species is limited. The inability of probiotic species to colonise the infant gut may mean that probiotic-associated modulation is short-lived. However, the reported benefits of probiotic prophylaxis may extend beyond colonisation and include competitive pathogen exclusion, changes to intestinal barrier function and immune modulation [8]. These positive effects on the developing microbiome may prove important for pre-term infants.

### Changes in probiotic-supplemented infants over time

Despite high levels of heterogeneity between individuals early in life, the infant microbiome generally follows a standardised colonisation process. As previously described, the choregraphed progression leads to typical microbial communities at different stages of the developmental process [42], as well as a reduction in heterogeneity and increased stabilisation with time [43]. Typically, aerobes dominate early on, defined by Dogra et al. as Cluster 1. Cluster 2 is characterised by higher levels of facultative anaerobes, especially those from Enterobacteriaceae, and Cluster 3 has higher abundances of strict anaerobes, particularly *Bifidobacterium*, in combination with lower abundances of aerobes like *Streptococcus* [42]. This previous work describes Cluster 3 as an end point, as once Cluster 3 is reached, subsequent samples from the same infant stay within this cluster. However, our cohort of infants had a significant reduction in *Bifidobacterium* after it rose to dominance.

The drop in *Bifidobacterium* and the lack of persistence of *B. bifidum* may be the result of confounding factors. Delayed colonisation or reduced counts of *Bifidobacterium* have previously been linked to caesarean section [42], and greater colonisation to breastfeeding [25]. The link to breastfeeding largely stems from the presence of human milk oligosaccharides in breastmilk, which are complex glycans that selectively nourish specific microbes [44, 45]. Without nourishment, microbes like *B. bifidum* may not persist. This may also explain why our work does not align with that of Abdulkadir et al. who observed persistence of *B. bifidum* following supplementation with Infloran^®^ and post discharge [14]. An important distinction between this study and theirs is that their entire cohort was breastfed, contrasting with only four infants in this study. Additive to this is that formula was introduced to all our infants’ post-discharge (Supplementary Table 1). Thus, diet may be an important factor for sustaining colonisation, and a modifiable factor that could encourage the long-term persistence of probiotic species in preterm infants.

*L. acidophilus* was not present post-discharge. Previous work has also struggled to isolate both the species and genus in supplemented infants [14, 15, 46]. However, unlike *B. bifidum*, this may not be a result of diet. Yousuf et al. who also observed an inverse relationship of *Lactobacillus* and antibiotic exposure, suggests that this is due to *Lactobacillus* being a coloniser of the small intestine and less likely to be found in faecal samples [15]. In addition, in our longitudinal analysis, we observed that the genus does not consistently establish itself in the gut during admission/prophylaxis, which is also supported by our previous work [17]. In this previous work, we suggest that this lack of colonisation could be the result of poor probiotic integrity. However, more work still needs to be done to provide conclusive evidence. Taken together, it is likely that the persistence of probiotic species and even bacterial community succession over the long term is determined by multiple environmental factors. Development of the gut microbiome in early life appears to mimic ecological primary succession, involving pioneer organisms which colonise the newly developed and relatively sterile habitat, changing the environmental conditions and thereby dictating succession through provision of niche conditions.

### Comparison of probiotic-supplemented infants and non-supplemented infants

Positive probiotic-associated modulation may not persist beyond discharge. Previous work suggests probiotic prophylaxis contributes to acute increases in bacterial diversity and abundance of known commensals, as well as a reduction in potential pathogens. This positive modulation may explain why probiotics have been demonstrated to significantly reduce the incidence of stage 2 or more NEC and LOS, albeit with some level of heterogeneity [47]. However, the modulation contributing to disease reduction appears to be temporary and may result from the limited persistence of probiotic species as previously discussed. Durack et al. observed that probiotics can temporarily correct for the delayed diversification associated with preterm infants, but that the inability of the probiotic species to engraft meant that these benefits are lost when probiotic prophylaxis is complete [48]. Additionally, Yousuf et al. demonstrated that probiotic exposure in preterm infants resulted in increased relative abundance of *Bifidobacterium*, but not *Lactobacillus*, potentially due to the colonisation in the small bowel of the latter genus [15]. However, this effect reduced over time, particularly at 5-month follow-up [15]. The cessation of probiotic prophylaxis may be why we do not see previously observed modulation persists.

Although lower diversity is associated with probiotic supplementation, it is unclear if probiotics are a driver of this low diversity. This is especially true when one considers the inability of the probiotic species to persist. Rather, these results may suggest that probiotics cannot correct for the lower diversity common to the most premature of infants. However, if probiotics are the causative factor, the drop in diversity may be a result of the restructuring of the microbial ecology, where the probiotic supports growth of a few specific taxa. Either way, whether this lower diversity will have significant consequences for these infants is not known, and beyond providing stability, greater diversity may also have limited benefits. Many of the benefits afforded by probiotic prophylaxis are likely to come through support of key taxa that possess invaluable functionality, or functional benefits provided through the probiotic species themselves during the supplementation period. This may be through the ability to combat pathogens by preventing adhesion to the mucosa [49] or through the production of short chain fatty acids [50]. This is not to say that diversity is not beneficial, just that the presence of key taxa may provide more benefits to infant health.

There were significant differences in the abundance of three species between the probiotic groups. Although it has been implicated in fatty liver disease, *Clostridium_M sp001517625* is a relatively un-studied species [51]. *Clostridium* species are of particular interest in maintenance of gut health and an imbalance in two of the species clusters (XIVa and IV) has been implicated in the development of ulcerative colitis and overall gut health, due to its involvement in metabolism of bile acids and generation of short-chain fatty acids [50]. *Flavonifractor plauti* on the other hand is considered a common inhabitant of the human gut microbiome, but its role/significance is unclear. The species has been implicated in both beneficial and pathogenic roles, being linked to reduced Th2 immune responses in mice [52], potentially through catechin metabolism, and to colorectal cancer [53], potentially acting through its capacity to degrade beneficial flavonoids. Lastly, *Alistipes finegoldi* is also thought to be a common inhabitant of the gut microbiome, albeit at lower levels relative to other Bacteroidetes. In terms of the species pathogenicity, there is contrasting evidence. The species has been suggested to be protective against some diseases, such as liver fibrosis and colitis, but it has also been implicated as a pathogen in colorectal cancer and depression [54]. Thus, although significant differences in taxa exist, the consequences are again indeterminant.

Despite the apparent limited long-term benefits in microbial modulation, the acute modulation observed previously during supplementation may provide lasting benefits. Microbial perturbations, including lower diversity, have been consistently associated with disease. This includes obesity [55], metabolic syndrome [56], Crohn’s disease and ulcerative colitis [57, 58], multiple sclerosis [59] and more. However, equally important to note is the effect on the development of both innate and adaptive immune function [60], as perturbations in the gut microbiome have also been shown to have long-lasting metabolic and immunological dysregulation [61]. Delayed gut microbial diversification over the first year of life, along with altered composition and metabolic function, is significantly associated with a greater risk of atopy and asthma development in childhood [48]. Thus, the early-life gut microbiome, colonising a relatively sterile habitat, influencing the developing ecosystem, and in turn, immune and physiological conditions, may have the greatest impact on long-term health.

## Limitations

This work has limited statistical power and was unable to account for all known microbial covariates due to its small sample size. As stated in the methods, the recruitment and collection protocol involved contacting parents/guardians at home and relying on their involvement for the collection and postage of stool samples. This proved too much of a burden for a demographic of people who have limited incentive to be involved in the project and are dealing with the stresses of being a new parent. We recommend that future studies take this into consideration during study design and either have greater involvement in the collection process or target a larger group to ensure adequate sample size.

## Conclusion

Probiotic-supplemented preterm infants are protected from infectious diseases during their stay in the NICU; however, this study suggests that the prophylactic probiotic species from Infloran^®^ do not persist 18 months–2 years post discharge. The implications of this are unclear. While probiotic-supplemented infants showed a healthier microbiome at discharge compared to other infants who did not receive probiotic supplementation, probiotic-supplemented infants had lower diversity in their gut microbiome at 18 months to 2 years of age. The small sample size reduces the certainty of this result. Nonetheless, with the emergence of a significant body of literature implicating the early gut microbiome in immune system development, it is unclear if lower diversity at this age would have significant implications.

## Supplementary Information

Below is the link to the electronic supplementary material.

Supplementary file1 (DOCX 25 KB)

Supplementary file2 (CSV 104 KB)

Supplementary file3 (DOCX 139 KB)

## Data Availability

The sequencing dataset generated and/or analysed during the current study is available through the International Nucleotide Sequence Database Collaboration at the *National Center for Biotechnology Information (NCBI)* repository; BioProject IDs: PRJNA751712, PRJNA687291 & PRJNA805057.

## Abbreviations

ASV: Amplicon sequence variant
B: Bifidobacterium
BH: Benjamini-Hochberg
DNA: Deoxyribonucleic acid
EC: Enzyme commission
EC: Enzyme commission number
HMO: Human milk oligosaccharide
L: Lactobacillus
LoS: Late-onset sepsis
MetaCyc: Metabolic pathway database
MPA: Metagenomics analysis platform
NEC: Necrotising enterocolitis
NICU: Neonatal intensive care unit
NMDS: Non-metric multidimensional scaling
NQLD: North Queensland
PCoA: Principal coordinate analysis
PCR: Polymerase chain reaction
PERMANOVA: Permutational analysis of variance
ROP: Etinopathy of prematurity
rRNA: Ribosomal ribonucleic acid
SCN: Special care nursery
TCDB: Membrane transport proteins
TSS: Total sum scaling
THHS: Townsville Hospital and Health Service

## Code availability

The analysis workflow, both the bioinformatics pipeline and statistical analyses, can be found at JacobAFW/Long_term_effects_of_probiotics (github.com).
