# Supplementary material for: Exploring the long-term colonisation and persistence of probiotic-prophylaxis species on the gut microbiome of preterm infants: a pilot study
Source: Eur J Pediatr. 2022 Jul 7;181(9):3389–400. doi: 10.1007/s00431-022-04548-y (PMC9395480; doi:10.1007/s00431-022-04548-y)
Supplement: Supplementary file 1 — Supplementary file1 (DOCX 25 KB) [file 431_2022_4548_MOESM1_ESM.docx]

## Longitudinal analysis using 16S amplicon sequencing data on a subset of the cohort who had samples collected as part of previous work

| Categorical Variables | | |
| --- | --- | --- |
| **Variables** | **Levels** | **Count** |
| **Diet during admission** | Combination | 5 |
|  | Breastmilk | 1 |
| **Diet post-discharge** | Combination | 5 |
|  | Breastmilk | 1 |
| **Delivery** | Vaginal | 0 |
|  | Caesarean | 6 |
| **NEC** | Yes | 0 |
|  | No | 6 |
| **Sepsis** | Yes | 0 |
|  | No | 6 |
| **Antenatal antibiotics** | Yes | 1 |
|  | No | 5 |
| **Neonatal antibiotics** | Yes | 6 |
|  | No | 0 |
| **Chorioamnionitis** | Yes | 0 |
|  | No | 6 |
| **Preeclampsia** | Yes | 1 |
|  | No | 5 |
| **Maternal Diabetes** | Yes | 0 |
|  | No | 6 |
| **Continuous Variables** | | |
| **Variable** | **mean/median** | |
| **Gestational age at birth** | 28.6$\pm0.5$ | |

*Table 1: Full demographic/clinical data for infants that underwent 16S metabarcoding.*

**Beta Diversity**

|  | DF | Sum of squares | R2 | F | Pr(>F) |
| --- | --- | --- | --- | --- | --- |
| Timing of collection | 2 | 2.26 | 0.35 | 4.45 | <0.001 |
| Diet | 1 | 0.31 | 0.05 | 1.20 | 0.29 |
| Residual | 13 | 3.30 | 0.52 | NA | NA |
| Total | 16 | 6.38 | 1 | NA | NA |

*Table 2: results of permutational ANOVA.*

|  | R2 | Pr(>r) |
| --- | --- | --- |
| Timing of collection | 0.85 | <0.001 |
| Diet | 0.18 | 0.09 |

*Table 3: results of envfit analysis.*

**Alpha Diversity**

| Variable | Chisq | Df | Pr(>Chisq) |
| --- | --- | --- | --- |
| Timing of collection | 116.83 | 2 | < 0.001 |
| Diet | 0.10 | 1 | 0.79 |

*Table 4: ANOVA results from a generalised linear mixed effects model on Shannon Index diversity.*

| Contrast | Estimate | SE | P |
| --- | --- | --- | --- |
| Admission – Discharge | -0.71 | 0.25 | < 0.01 |
| Admission – Post-discharge | -3.02 | 0.29 | <0.001 |
| Discharge – post-discharge | -2.31 | 0.27 | <0.001 |

*Table 5: Tukey’s pairwise comparison from a generalised linear mixed effects model on Shannon Index diversity.*

| Variable | Chisq | Df | Pr(>Chisq) |
| --- | --- | --- | --- |
| Timing of collection | 513.55 | 2 | < 0.001 |
| Diet | 0.04 | 1 | 0.84 |

*Table 6: ANOVA results from a generalised linear mixed effects model on richness.*

| Contrast | Estimate | SE | P |
| --- | --- | --- | --- |
| Admission – Discharge | -15.45 | 8.70 | 0.18 |
| Admission – Post-discharge | -203.13 | 10.10 | <0.001 |
| Discharge – post-discharge | -187.66 | 9.21 | <0.001 |

*Table 7: Tukey’s pairwise comparison from a generalised linear mixed effects model on richness.*

**Taxonomic Abundance**

| Comparison | Lfc | Lfc SE | P-adj | Species |
| --- | --- | --- | --- | --- |
| Admission – Discharge | -6.80 | 1.72 | <0.001 | *Streptococcus* |
| Admission – Post-discharge | -7.28 | 1.96 | <0.001 | *Streptococcus* |
| Discharge – Post-discharge | 4.02 | 0.91 | <0.001 | *Bifidobacterium* |
| Admission – Post-discharge | 3.52 | 1.00 | <0.001 | *Bifidobacterium* |

*Table 8: DESeq2 differential abundance testing.*

# Cross-sectional analysis using shotgun metagenomics data

**Beta Diversity**

|  | DF | Sum of squares | R2 | F | Pr(>F) |
| --- | --- | --- | --- | --- | --- |
| Probiotics | 1 | 0.29 | 0.06 | 1.01 | 0.4 |
| Delivery | 2 | 0.65 | 0.13 | 1.14 | 0.24 |
| Diet | 1 | 0.24 | 0.05 | 0.85 | 0.6 |
| Residual | 13 | 3.71 | 0.75 | NA | NA |
| Total | 17 | 4.92 | 1 | NA | NA |

*Table 10: results of permutational ANOVA.*

|  | R2 | Pr(>r) |
| --- | --- | --- |
| Probiotics | 0.01 | 0.88 |
| Delivery | 0.11 | 0.88 |
| Diet | 0.02 | 0.88 |

*Table 11: results of envfit analysis.*

**Alpha Diversity**

| Variable | Chisq | Df | Pr(>Chisq) |
| --- | --- | --- | --- |
| Probiotics | 5.28 | 1 | < 0.05 |
| Delivery | 0.69 | 2 | 0.71 |
| Diet | 0.06 | 1 | 0.80 |

*Table 12: ANOVA results from a generalised linear mixed effects model on Shannon Index diversity.*

| Contrast | Estimate | SE | P |
| --- | --- | --- | --- |
| Probiotics: No - Yes | 0.63 | 0.27 | < 0.05 |

*Table 13: Tukey’s pairwise comparison from a generalised linear mixed effects model on Shannon Index diversity.*

| Variable | Chisq | Df | Pr(>Chisq) |
| --- | --- | --- | --- |
| Probiotics | 6.53 | 1 | < 0.05 |
| Delivery | 0.12 | 2 | 0.94 |
| Diet | 0.26 | 1 | 0.60 |

*Table 14: ANOVA results from a generalised linear mixed effects model on richness.*

| Contrast | Estimate | SE | P |
| --- | --- | --- | --- |
| Probiotics: No - Yes | 38 | 14.87 | < 0.05 |

*Table 15: Tukey’s pairwise comparison from a generalised linear mixed effects model on richness.*

**Taxonomic Abundance**

| Variable: base level | Lfc | Lfc SE | P-adj | Species |
| --- | --- | --- | --- | --- |
| Probiotics: Yes | 29.09 | 2.59 | < 0.01 | Clostridium_M sp001517625 |
| Probiotics: Yes | 18.66 | 1.95 | < 0.01 | Flavonifractor plautii |
| Probiotics: Yes | -16.02 | 2.53 | < 0.01 | Alistipes finegoldii |

*Table 15: DESeq2 differential abundance testing.*
