# Supplementary material for: Exploring the long-term colonisation and persistence of probiotic-prophylaxis species on the gut microbiome of preterm infants: a pilot study
Source: Eur J Pediatr. 2022 Jul 7;181(9):3389–400. doi: 10.1007/s00431-022-04548-y (PMC9395480; doi:10.1007/s00431-022-04548-y)
Supplement: Supplementary file 3 — Supplementary file3 (DOCX 139 KB) [file 431_2022_4548_MOESM3_ESM.docx]

**PARENTAL INFORMATION SHEET and CONSENT FORM**

***PROTOCOL NAME: Exploring the Long-Term Effects of Probiotic Supplementation in Premature Infants***

***INVESTIGATORS:***

**Dr Donna Rudd - James Cook University**

**A/Prof Yoga Kandasamy - Townsville Hospital & James Cook University**

**Jacob Westaway - James Cook University**

**Dr Roger Huerlimann - Okinawa Institute of Science and Technology Graduate University**

1. **Introduction**

You are invited to give consent for your baby to take part in the *Exploring the Long-Term Effects of Probiotic Supplementation in Premature Infants study*. Before you decide whether to allow your baby to participate or not, it is important for you to understand why this research is being done and what it will involve. This information sheet tells you about the research study. Please take the time to read through this information and discuss it with others if you wish. Ask questions about anything you don’t understand or would like to know more about.

This study is entirely voluntary (your choice). If you decide you would like your baby to take part in this research study, you will be asked to sign the consent form. You will be given a copy of this information sheet and consent form to keep.

Thank you for your interest and for taking the time to consider whether you wish your baby to take part in the study.

1. **What is the purpose of this research?**

Everybody has microscopic organisms living in and on their body called their microbiota. These microorganisms live on your skin, in your gut and other organs, interacting with the environment and providing beneficial effects to the human body. Previous research has looked at the development of gut microorganisms in babies, where they come from and how they affect the person’s health and well-being for the rest of their life. When this development does not happen properly, it may cause disease, such as sepsis, asthma and coeliac disease. Which is why many preterm infants now receive probiotics as standard care. The infant microbiota and its development following birth may be an important factor for keeping us healthy. However, while the development of the preterm faecal microbiota and effects of probiotic supplementation have been studied, long-term studies on microbiota development and probiotics are limited.

The purpose of this study is to investigate the faecal microorganisms of pre-term (premature) babies born before 32 weeks and those born after 32 weeks. We will supply you with a collection kit and postal supplies and ask that you collect their faeces at home, and post the sample back to us. We would like to see if treatment put in place by your care giving team whilst admitted has changed the baby’s microorganisms.

It is possible that the results of this study will help us find factors we can modify or preventative measures to make sure the baby’s faecal microbiota develops normally over early life.

This research study is being undertaken at the Townsville Hospital Neonatal Intensive Care Unit working in collaboration with James Cook University (JCU).

The results of this research study will be used by Dr Donna Rudd (Supervisor) and Mr Jacob Westaway, as results for a PhD thesis (JCU). These results will also be used by the research team for publication and leverage of research funding.

1. **Who can participate in this research?**

Your baby can participate in this study if

- Your Baby has been identified as being suitable by the Neonatal Intensive Care specialist
- They were born before 32 weeks and were administered probiotics as part of their on-going treatment in the Neonatal Intensive Care Unit,
- or if your baby was born after 32 weeks and was admitted to the Special Care Nursery

1. **What does participation in this research involve?**

The care for your baby will be provided as normal by your doctors, nurses and health care givers. If you agree to take part in this study, we will ask you to complete and sign a consent form.

We ask that you use the provided sample collection and postage kit to collect your infants faeces at their next home bowel movement, no other samples are required.

A member of the research team will collect information about the pregnancy and your baby’s birth from your medical notes.

**5 Does my baby have to take part in this study?**

Participation in this study is voluntary (your choice). It is completely up to you if you wish to participate or not.

If you decide to allow your baby to participate (or not), it will not affect the care that your baby will receive now or in the future, and will not affect your relationship with the staff caring for your baby.

If you do decide to allow your baby to participate you can still withdraw your baby from the study at any time and you don’t have to give a reason.

1. **What are the alternatives to participation?**

If you chose not to allow your baby to participate in the study your baby will receive normal care from the doctors and nurses.

1. **What are the possible benefits of taking part?**

We cannot guarantee or promise that you or your baby will receive any benefits from this study; however, these are some possible benefits. The results from this study may be used to inform treatment of babies in the future to maintain their faecal microbiota.

1. **What are the possible risks and disadvantages of taking part?**

This is a Non Invasive Clinical Research study. Only your baby’s stool (faeces) sample is collected for analysis. There is no change in the treatment of your baby.

1. **What will happen to my baby’s samples and results?**

All stool (faeces) samples will be sent to the James Cook University laboratory, and Microba, an industry partner for analysis of microbial content. Following this analysis the sample will be disposed of using appropriate protocols.

All data related to the study will be stored on the hospital’s information system as per normal protocol. Any other data will be stored without identifying information until publication of results.

Non-identifiable data may need be made available as a requirement of publication policies.

**10 Can my baby have other treatments during this research project?**

Yes. Your baby will continue to receive all the necessary treatment as decided by the neonatal doctors and nurses.

**11 What if I join the study but then change my mind and want to withdraw?**

Participation in this study is voluntary. You can withdraw from the study at any time and you do not have to give a reason. Please let a member of the research team know and they will get you to complete a “Withdrawal of Consent” form.

If you decide to no longer allow your baby to participate in the study no further information will be collected about you or your baby. You should be aware, however, that data collected up to the time you withdraw will be kept and will form part of the research study results.

A decision to withdraw from the study will not affect the care your baby will receive now or in the future and will not affect your relationship with the staff caring for you

**12 What happens when the research project ends?**

The results from this research study will be analysed. They will then be presented at conferences and professional forums and written up and submitted to medical journals to publish. They will also be written up in the format of a PhD thesis.

In any publication, presentation or report, information will be presented as summary data so no participant can be identified. Summary results of the data will also be provided to you by a letter from the researchers after publication, if you wish. Please note there is a significant delay between data collection and publication of the results.

**13 What will happen to information about my baby?**

All information collected about participants during the study will be kept strictly confidential. No material that could potentially identify any participant will be used in any report of this study. Your personal details will be held securely within the Townsville Hospital and only members of the research team will have access to them.

**14 Will I be paid for taking part in this study?**

There is no payment or incentive for participating in this study.

**15 Who is organising and funding the research?**

The study is being funded by Townsville Hospital and Health Service Study, Education and Research Trust Account (SERTA) Fund and organised discipline of Biomedicine at the College of Public Health, Medical and Veterinary Sciences at James Cook University.

**16 Who has reviewed the research project?**

All research in Australia involving humans is reviewed by an independent group of people called a Human Research Ethics Committee (HREC). The ethical aspects of this research study have been reviewed and approved by both the Townsville Hospital and James Cook University HREC.

**17 Further information and who to contact**

After you have read this information, a member of the research team will discuss the study with you again and answer any questions you may have. You will be given the opportunity to discuss your baby’s participation in this study with whomever you wish, such as your partner, other family, friends or your doctors and midwives providing your care. If you would like to know more at any stage, please do not hesitate to contact the research contact person below.

**Research Contact Person:**

Name: Dr Donna Rudd

Position: Associate Professor Biomedicine

Phone: (07) 47816262

Email: donna.rudd@jcu.edu.au

**This project has been reviewed and approved by the Townsville Hospital and Health Service Human Research Ethics Committee, and James Cook University Human Research Ethics Committee. For concerns relating to the conduct of this project contact either:**

**Townsville Hospital and Health Service Human Research Ethics Committee:**

HREC Chairperson

Phone: 07 4433 1440

Email: [TSV-Ethics-Committee@health.qld.gov.au](mailto:TSV-Ethics-Committee@health.qld.gov.au)

**James Cook University Connect Human Ethics:**

Human Ethics Officer

Phone: 07 4781 6575

Email: helen.griffiths@jcu.edu.au

**Thank you for taking the time to read this and consider being involved in the study.**

**You will be provided with a copy of this form to keep.**

###### PATIENT/PARTICIPANT CONSENT FORM

***PROTOCOL NAME:*** Exploring the Long-Term Effects of Probiotic Supplementation in Premature Infants

## INVESTIGATORS: Dr Donna Rudd, A/Prof Yoga Kandasamy Dr Roger Huerlimann, and Mr Jacob Westaway

1. The nature and purpose of the research project has been explained to me. I understand it and acknowledge that taking part in this study is voluntary.

2. I have been given an Information Sheet which explains the purpose of the study, the possible benefits, and the possible risks for my child.

3. I understand that my child may not directly benefit from taking part in the study.

4. I understand that, while information gained during the study may be published, my child will not be identified, and their personal results will remain confidential.

5. I understand that I can withdraw my child from the study at any stage and that it will not affect my child’s medical care, now or in the future.

6. I have had the opportunity to discuss taking part in this investigation with a family member or friend.

I wish to receive a copy of the summary results when they are available. YES NO

I can be contacted by email or phone (Please leave email addresses or phone number):

Please note there is a significant delay between data collection and publication of the results.

**NAME OF PARTICIPANT (GUARDIAN):**

**NAME OF PARTICIPANT (CHILD):**

**SIGNATURE:**

**DATED:**

I certify that I have explained the study to the patient and consider that she understands what is involved.

**NAME OF RESEARCH TEAM MEMBER:**

**SIGNATURE:**

###### PARENTAL WITHDRAWAL OF CONSENT FORM

***PROTOCOL NAME: Exploring the Long-Term Effects of Probiotic Supplementation in Premature Infants***

I hereby wish to WITHDRAW my consent to allow my baby to participate in the study described above. I understand withdrawal WILL NOT jeopardise or change my baby’s treatment or relationship with the staff at Townsville Hospital.

I understand that no further information will be collected about me or my baby, however, data collected up until now will be kept and will form part of the research study results.

**NAME OF PARTICIPANT:**

**SIGNATURE:**

**DATED:**
